# Evaluating the Suitability of Perfusion-Based PD Probes for Use in Altered Gravity Environments

**DOI:** 10.3390/bios15080478

**Published:** 2025-07-24

**Authors:** Madelyn MacRobbie, Vanessa Z. Chen, Cody Paige, David Otuya, Aleksandra Stankovic, Guillermo Tearney

**Affiliations:** 1 Harvard-MIT Division of Health Sciences and Technology (HST), Boston, MA 02139, USA; stankov@mit.edu (A.S.); gtearney@mgb.org (G.T.); 2 Department of Aeronautics and Astronautics, Masschusetts Institute of Technology, Cambridge, MA 02139, USA; 3 Wellman Center for Photomedicine, Massachusetts General Hospital, Boston, MA 02114, USA; dotuya@mgh.harvard.edu; 4 Biomedical Engineering, University of Waterloo, Waterloo, ON N2L 3G1, Canada; vzchen@uwaterloo.ca; 5 Space Exploration Initiative, MIT Media Lab, Cambridge, MA 02139, USA; cpaige@mit.edu; 6 Harvard Medical School, Boston, MA 02115, USA; 7 Center for Space Medicine Research, Massachusetts General Hospital, Boston, MA 02114, USA; 8 Department of Pathology, Massachusetts General Hospital, Boston, MA 02114, USA

**Keywords:** electrophysiology, parabolic flight, microgravity, potential difference

## Abstract

Measurable changes in electrophysiology have been documented in spaceflight, creating a pathway for disease genesis and progression in astronauts. These electrophysiology changes can be measured using potential difference (PD). A probe to measure PD was developed and is used clinically on Earth; this probe relies on fluid perfusion to establish an electrical connection to make PD measurements. The changes to fluid behavior in microgravity and partial gravity (including lunar and Martian gravity) drives the need to test this probe in a spaceflight environment. Here, we test the PD probe in a novel nasal cavity phantom in parabolic flight, simulating microgravity, lunar gravity, Martian gravity, and hypergravity conditions across 37 parabolas. The results are evaluated across gravity conditions using the Wilcoxon Rank Sum test. We record no statistically significant difference in probe PD measurements in 1 g, microgravity, lunar gravity, and hypergravity (approximately 1.8 g) conditions, reaching a NASA Technology Readiness Level 6. Martian gravity findings are inconclusive. Perfusion-based PD probes are therefore successfully demonstrated for use in spaceflight operation in microgravity, lunar gravity, and hypergravity environments; this establishes a foundation for moving towards the in-space testing of perfusion-based probes in astronauts.

## 1. Introduction

NASA’s upcoming Artemis missions and Moon to Mars plans indicate a renewed interest in human exploration of the Moon and beyond [1]. These plans come with extended durations in space and increased distances from Earth, which introduces a variety of new medical risks. Exposure to microgravity leads to physiological changes that put astronauts at risk of developing symptoms which adversely affect performance during a mission, and can also have long-term health impacts following return to Earth [2]. There is a growing need for medical devices that can monitor, diagnose, and treat in-space medical conditions to enable astronauts to venture further from Earth.

Ion channel-dependent processes are one physiologic function that changes in altered gravity environments; this has been demonstrated in spaceflight, and also in space analogs like parabolic flight [3,4,5,6]. The demonstrated shift in ion transport across a membrane creates measurable changes in parameters such as potential difference and resistance. Electrophysiology measurements are therefore needed in spaceflight environments to further elucidate the mechanisms underlying these changes and monitor the physiologic state in astronauts throughout a mission.

**Figure 1:**
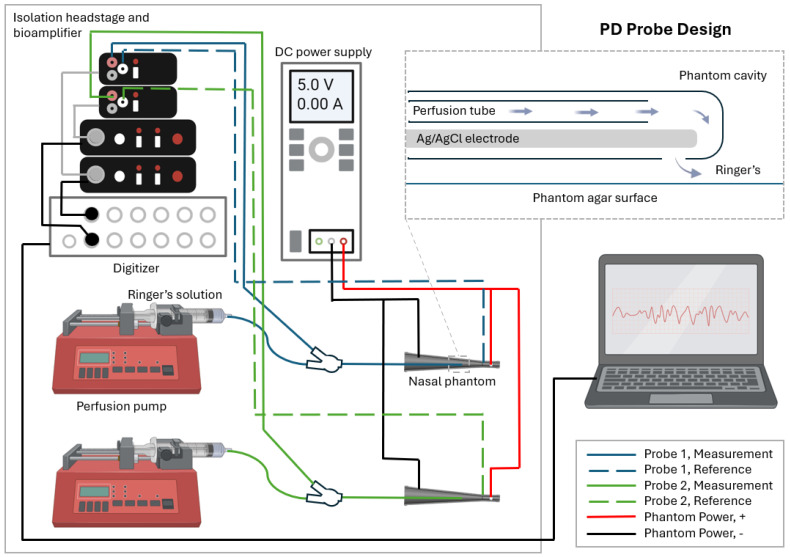
The flight setup contains two complete PD systems and phantoms.

Potential difference (PD) is used clinically for the investigation of ion channel-related diseases such as cystic fibrosis, which result in electrophysiological changes that can be measured at the tissue surface. An ultra-small, minimally invasive probe to measure PD in vivo has been developed and demonstrated in intestinal and respiratory epithelium [7,8]. This probe, shown in Figure 1, utilizes a novel Ag/AgCl measurement electrode in combination with a commercially available ECG skin patch electrode as a reference. The measurement probe features a 1.2 mm outer diameter (OD) sheath, which encompasses the electrode, a 350 μm perfusion tube, and an adhesive seal to create a small electrode cell at the tip of the probe. Ringer’s solution (Thermo Fisher Scientific, Cincinnati, OH, USA) is perfused continuously through a perfusion port to establish an ionic bridge between the electrode and the target measurement surface.

Benchtop testing can be carried out with an agar phantom for rapid iteration and development prior to introducing an animal or clinical model. The measured PD value is connected to the local ionic concentration at the site of respective probes by the Goldman–Hodgkin–Katz voltage equation with the movement of ions following the rate of reaction between the probe and the agar, such that [8]

(1) $$PD=E^{0}-\frac{RT}{F}ln\frac{{\left[Cl^{-}\right]}_{ref}\gamma_{{\left[Cl^{-}\right]}_{ref}}}{{\left[Cl^{-}\right]}_{ref}\gamma_{{\left[Cl^{-}\right]}_{ref}}-Q_{0}exp\left(BV\gamma_{{\left[Cl^{-}\right]}_{ref}}\right)}$$

with

(2) $$B=\frac{(1-\alpha )FV}{RT}$$

where *R* is the gas constant, *T* the absolute temperature of the test medium, *F* the Faraday’s constant, *Cl*^−^ denotes chloride anions, $Q_{0}$ is an arbitrary constant that can be determined empirically, *ref* denotes the reference probe, γ represents ionic activity at the corresponding ion at the given ionic concentration, and *V* is the applied voltage. Applying a voltage across the agar in the phantom therefore establishes a differential in the ionic concentration near the reference probe. For a simple agar composition including KCl, the chloride ions are attracted to the positive electrode and the ions undergo oxidation to form chlorine molecules. The released electrons then flow through the probe’s external measurement circuit.

Gravity plays a role in driving physical processes that influence chemical kinetics, such as diffusion, the formation of concentration gradients, and driving collisions between molecules. The reaction rate in a much stronger gravitational field is slower than the reaction rate in a much weaker gravitational field, with constant temperature and pressure due in part to the higher kinetic energy of molecules in a weaker gravitational field [9]. However, the molecular interactions underlying the chemical reactions do not rely on gravitational effects. For differences between 1 g and microgravity, the molecular processes have been shown to dominate reaction rates [10] and, as such, the role of gravity is negligible in the chemical kinetics underlying our PD probe’s interaction with a phantom across gravity environments.

Changes in fluid behavior with altered gravitational field strength are well documented [11,12,13]. The PD probe relies on fluid perfusion for electrode measurement. This probe uses slow, continuous perfusion at 2.5 mL/h to maintain laminar flow while ensuring a constant supply of ions for the electrode oxidative measurement. The Bond number is a dimensionless number that describes the ratio of gravitational to surface tension force:

(3) $$B_{0}=\frac{\Delta \rho gL}{\gamma }$$

where Δρ represents the difference in the density of the phase interface, *g* the gravitational acceleration, *L* the characteristic length such as the circumference of the advancing fluid section, and γ the surface tension of the fluid. On planetary surfaces like the Earth, Moon, and Mars, the Bond number indicates gravity-dominated fluid behavior, while microgravity environments exhibit surface tension-dominated fluid behavior [14]. The use of fluid perfusion in PD probes therefore needs testing in altered gravity environments to determine whether functionality is impacted in the change from gravity-dominated to surface tension-dominated fluid behavior.

## 2. Materials and Methods

### 2.1. Parabolic Flight

**Figure 2:**
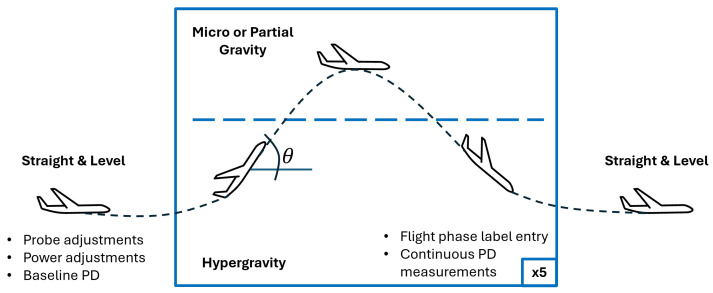
Overview of parabolic flight and the in-flight procedures used in this experiment. The aircraft pitch θ changes throughout the parabola and determines the resulting net gravitational environment. Notably, the microgravity phase of flight begins prior to the nose-down portion; the acceleration on the aircraft is downward as it nears the top of its arc, creating a net microgravity force. Hypergravity occurs as the aircraft pulls out of the dive and initiates a climb to regain altitude for the next reduced gravity phase.

Parabolic flight is a primary Earth-based spaceflight simulation that is a key step in hardware development for space, providing a relevant environment for device testing to reach a Technology Readiness Level (TRL) of up to 6 [15]. The mathematics of parabolic flight are well documented, and parabolic flight is a widely used spaceflight analog for fluids, human factors, and physiology experiments, among others [16,17]. During the microgravity phase of the flight, the aircraft pitch θ is such that the acceleration along the aircraft vertical is

(4) $$\sum F_{z}=-g\cos\theta$$

resulting in a net microgravity force. Partial gravity (including lunar and Martian gravity) is achieved by reducing the pitch angle. Each parabola in a parabolic flight alternates a reduced (micro- or partial) gravity arc with a hypergravity arc of approximately 1.8 g to re-set the aircraft trajectory for the next parabola. An overview of parabolic flight is shown in Figure 2.

In this experiment, a total of 37 microgravity and partial gravity parabolas were conducted via Zero-G’s G-Force One, a specially modified Boeing 727 (Zero Gravity Corporation, VA, USA). Each parabola provides approximately 15–20 s of altered gravity. These occurred in sets of five parabolas, with about 2 min of straight and level flight between sets.

### 2.2. Statistical Approach

An a priori power analysis was conducted for each of the gravitational conditions using G*Power version 3.1.9.7 for sample size estimation [18]. Ground testing was conducted with the probe and phantom setup to record baseline PD values in the contact and no-contact conditions. An α error probability of 0.05 and 0.95 power in a two-tail bivariate model results in a sample size of three parabolas for each gravity environment. The pre-flight plan included a minimum of 20 total parabolas, with each gravity level planned for a minimum of two parabolas. As such, two complete phantom and PD probe setups were included in the flight configuration to reach significance and provide an additional margin for data collection across all gravity conditions investigated.

### 2.3. Flight Procedures

Per Zero-G procedures, all major hardware components were loaded into the aircraft following the successful completion of a flight readiness review 1 day prior to flight. The phantoms and syringes containing fluids were loaded the morning before the flight to allow for overnight storage in Ringer’s solution, to maintain the agar layer. The flight configuration of the experiment setup is shown in Figure 1. Perfusion pumps began operation on the ground prior to takeoff in order to remove bubbles from the probe in a standard 1 g environment.

Once in straight and level flight, prior to beginning the parabolas, data collection in LabChart (ADInstruments, Sydney, Australia) was initialized to record a 0 V flight baseline. Each gravity transition was recorded in real time by the experiment operator in LabChart, and any probe placement adjustments were made during the straight and level flight between parabola sets. The device was allowed to continuously run to record PD values throughout the flight, with either 0 V or 5 V applied to the phantom in alternating parabola sets. Baseline straight and level flight was recorded between each set. In-flight procedures during each flight phase are summarized in Figure 2. Hypergravity was included in all sets between parabolas. Sets 5–7 were training parabolas, used to train new pilots in Zero-G flight operations; additional variations may have occurred as an artifact of turbulence due to training.

## 3. Nasal Phantom Design and Development

**Figure 3:**
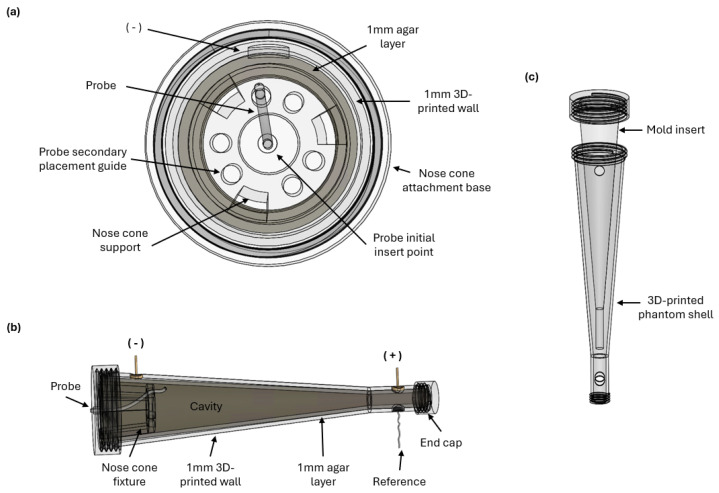
The nasal phantom design. (**a**) Cross-sectional view of the phantom with the measurement probe insert, or nose cone, fixture. (**b**) Phantom side view in full test configuration, with agar layer, power supply inputs, and reference attachment. (**c**) Phantom shell with corresponding mold insert, used in the agar fill procedure and removed to create the cavity.

The PD probe used in this study underwent clinical testing in the nasal cavity [8]. A novel nasal cavity phantom (Figure 3) was engineered to simulate the anatomical and electrical characteristics of the human nasal cavity, facilitating in-flight measurements without human or animal subjects.

### 3.1. Phantom Geometry

The phantom design emulates key features of the nasal cavity geometry that are relevant for PD probe operation. The PD probe is intended for astronaut self-operation in space; for this, the astronaut would insert the device into the nose and, as such, PD probe placement somewhere along the epithelium inside the nasal cavity is assumed, without specifying an exact location. The piriform aperture is the largest portion of the human nasal cavity inferior to the nasal bones and is the target area for device placement. It has a mean lower width of 23.1 mm at the nose opening, which narrows to 15.7 mm at the upper end [19]. A simplified model was developed to reflect these diameters and the natural tapering of the nasal cavity, to enable a qualitative evaluation of probe fit, placement, and fluid motion in addition to the quantitative PD measurements. The model was 3D-printed using a J55 Prime PolyJet printer (Stratasys, MN, USA) with an 18.75 µm resolution to print within the tight tolerances required for cap threading. A 1 mm VeroClear (Stratasys, MN, USA) phantom wall thickness was chosen to balance structural integrity with the need for probe visibility during the experiment. This is shown in Figure 3a,b.

**Figure 4:**
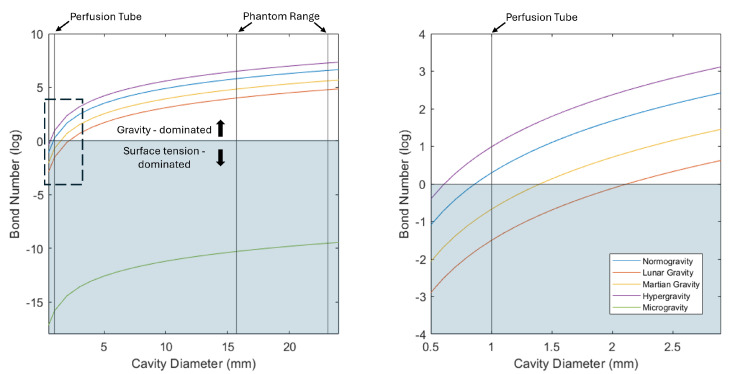
Calculated effect of cavity geometry on the dominant fluid force in different gravitational environments. The dashed line denotes the region of the plot shown on the right.

Equation (3) can be applied to both the fluid perfusion out of the probe and the fluid motion within the cavity of the nasal phantom to understand the fluid behavior within each test environment. The characteristic length *L* changes depending on the location of the fluid within our test setup, such that the circumference of the advancing fluid section varies between the probe’s perfusion tube (inner diameter 1 mm) and the largest portion of the phantom cavity (inner diameter 23.1 mm). Figure 4 shows the relationship between cavity geometry and the resulting Bond number across gravitational test environments. Notably, gravity-dominated fluid behavior is expected in every test environment other than microgravity once the fluid exits the perfusion tube and enters any portion of the phantom cavity. Due to the small diameter of the perfusion tube, surface tension-dominated fluid behavior is expected within the tube in Martian, Lunar, and microgravity.

The expected fluid behavior inside the phantom cavity in each gravitational environment was confirmed by qualitative observation in flight. The perfused fluid consisted of Ringer’s solution mixed with red food dye (Whole Foods Market, TX, USA) to ensure visibility in both the perfusion tube and phantom cavity. This also acted as a safety feature for the parabolic flight test, enabling rapid identification if a fluid leak occurred.

### 3.2. Agar Layer

An agar layer was made to closely mimic the dielectric properties of nasal tissues when interfaced with a PD probe, following the relationship described in Equation (1). A 3 M agar–Ringer’s mixture was made by boiling 3 g agar (Sigma-Aldrich, St. Louis, MO, USA) in 100 mL of Ringer’s solution to 100 °C. The 3D-printed phantom shell and a corresponding mold insert, as shown in Figure 3c, were filled with the agar solution. Upon cooling, the mold insert was removed.

Optimizing agar thickness to achieve the thinnest possible layer to enhance probe visibility, while still maintaining the desired shape, was a critical aspect of the phantom’s development for this experiment. Several trials were conducted with molds designed to leave agar layers of 1 mm, 2 mm, and 3 mm thicknesses. All trials held their shape, with the agar drying to a translucent finish. As such, the 1 mm thickness was selected for visibility. The 3D-printed phantom was then sized so that the inner cavity met the desired 23.1 and 15.7 mm dimensions inside the agar layer.

### 3.3. Power Supply and Reference Probe

To measure changes in PD using the phantom, a voltage was applied to the agar layer. This necessitated the addition of two leads to attach the positive and negative inputs of a DC power supply. The 3D-printed phantom included two holes along the same axis, at the large and small ends (distal and proximal piriform aperture approximations, respectively) of the phantom shell. A gold earring stud base was attached to the hole with epoxy, making sure to leave the metal base exposed inside the phantom. The agar was then filled, establishing direct contact between the conductive metal base and the agar. The power supply was attached via alligator clip to the stick post of the earring base, enabling a power modulation that could be measured with the PD probe.

The PD probe requires a reference in addition to the measurement probe. In our phantom, this was accomplished via an Ag/Cl lead wire embedded in the agar layer. The Ag/Cl wire was inserted into a hole in the narrow end of the phantom, opposite the positive electrode lead, and looped to hold its position against the wall of the phantom cavity. Upon the addition of the agar, the reference lead was embedded in the agar without being exposed to the interior cavity. An alligator clip could be used to connect the lead wire to the PD system as the reference measurement. The power supply and reference probe attachments are included in Figure 3b.

### 3.4. Measurement Probe Insert Fixture

PD measurements depend on the contact between the electrode and the measurement surface, facilitated by fluid perfusion. The probe must therefore maintain its placement against the agar surface; to this end, an insert was designed to guide the probe towards the agar layer and hold it in place throughout the flight. This insert utilized a “nose cone” fixture to route the probe to the area around the positive electrode’s location, with multiple hole options to allow for adjustments, as shown in Figure 3a. This alignment is critical for minimizing variations in resistance to obtain accurate PD readings. It also ensures that the probe’s orientation remains constant, isolating the effects of gravity on fluid perfusion for probe contact and measurement accuracy.

## 4. Results

### 4.1. Ground Validation of Tunable PD

**Figure 5:**
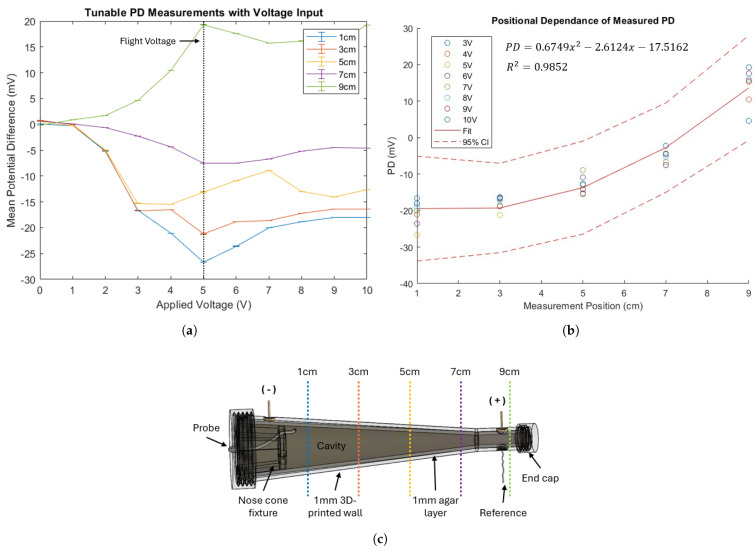
Benchtop (1 g) demonstration of the nasal phantom for tunable, position-dependent PD measurements. (**a**) The recorded PD tunability based on input voltage at various measurement positions inside the phantom cavity, indicating the ability to select an input voltage to record within a desired range for phantom simulation. (**b**) Recorded PD varies with probe placement following a quadratic relationship, as predicted by Coulomb’s equation, with two point charges. (**c**) Location of the probe placement in the nasal phantom.

The nasal phantom was tested with the PD probe in a normal 1 g environment for ground validation prior to flight testing. Input voltages ranging from 0–10 V were applied to the agar layer and allowed to stabilize for two minutes. The PD probe was then used to record measurements inside the phantom cavity, taking an average measurement over a one-minute interval. Because our intended device operation specifies placement within a range inside the nasal cavity, measurements were taken at different positions inside the phantom. The probe was used to measure PD at 1, 3, 5, 7, and 9 cm positions from the negative voltage input location, as shown in Figure 5c.

The results confirming PD’s tunability in the nasal phantom are shown in Figure 5a. These informed the selection of the 5 V applied voltage used in the parabolic flight test, given the range of recorded PD measurements based on probe position. The positional dependence of the recorded PD is evaluated in Figure 5b; the results follow the expected $x^{2}$ dependence indicated by Coulomb’s equation for a setup with two point sources, which, in this case, are the positive and negative power supply inputs. The results confirm the utility of the nasal phantom for tunable, position-dependent PD measurements in a 1 g environment.

### 4.2. PD Probe Operation in Parabolic Flight

**Figure 6:**
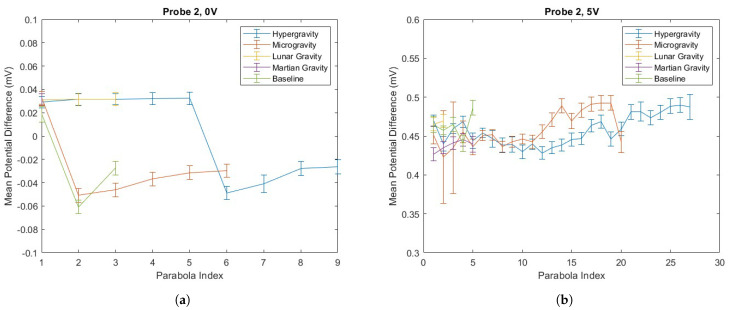
Recorded PD variations in altered gravity environments. (**a**) Probe 2, 0 V; (**b**) Probe 2, 5 V.

PD was recorded across 37 parabolas, alternating intervals of reduced gravity and hypergravity, as described in Section 2.1. Probe 1 became dislodged from its placement within the phantom between sets of parabolas, resulting in fewer total parabola samples being collected with Probe 1. Due to the incomplete dataset for Probe 1, the analysis that follows focuses on Probe 2. The results from Probe 1 are available in the Supplementary Materials. Recorded PD values are averaged within each parabola section, with the mean and standard deviation for each shown in Figure 6.

An inherently unequal number of samples were recorded within each parabola due to variations in parabola duration, and an unequal number of each parabola type. As such, the nonparametric Wilcoxon rank sum test was used to evaluate whether there is a difference in PD measurement across gravity environments. The significance criterion was set at *p* < 0.05, such that values below this threshold indicate a statistically significant difference between measured PD values. Effect size is calculated as the mean difference in PD measurement, and reported as an absolute value. The results are shown in Table 1.

## 5. Discussion

### 5.1. Perfusion Probes in Altered Gravity

The PD values recorded in flight are in the range of 0.4–0.5 mV, which is lower than the range measured in the ground validation studies. This can be accounted for by procedural differences; the ground validation studies occurred shortly following agar was filled in the phantom, while the parabolic flight measurements followed a 48 h period of phantom storage, submerged in Ringer’s solution. This, combined with use of red dye in the perfusate for in-flight visualization, changes the ion concentration at the measurement site. Ringer’s solution contains approximately 110 mM of chloride anions, while the agar phantom was designed with a 3 M concentration. We therefore expect the 48 h storage period in which phantoms are submerged in Ringer’s solution to enable the diffusion of chloride anions, which would reduce the concentration of the chloride ions in the agar. This would then reduce the PD that would be measured in the flight phantom, following the relationship described in Equation (1). This is confirmed by the measured PD in the flight phantoms (0.4–0.5 mV at 7 cm) being lower compared to the ground validation phantoms (5 mV at 7 cm) for the same applied voltage. The ground validation studies demonstrate that the nasal phantom can be used to simulate physiological PD and correlate this measurement to both input voltage and probe placement.

The PD probe’s suitability for use across gravitational environments is successfully demonstrated in the parabolic flight tests. PD varied by less than 0.06 mV across gravity environments, as reported in Table 1. In the statistical test used, a successful test is defined as one where the significance criterion is not met, such that *p* > 0.05. This indicates that there are not statistically significant differences in probe functionality between operation in 1 g and in altered gravity. Table 1 shows this to be the case for most test pairings, with the exception of 3, involving Martian gravity. However, the Martian gravity parabolas included in this analysis primarily occurred at the beginning of the initial training parabolas for new Zero-G pilots and may not be indicative of the typically consistent gravitational environment experienced throughout the parabola. In other words, variations in the gravitational environment introduced during these parabolas are expected to influence this result and create an artificial difference in PD measurement. While there were also training parabolas of lunar and microgravity, these were balanced by parabolas prior to training that were included in the analysis.

Accelerometer data was recorded for the flight by the MIT Media Lab Space Exploration Initiative and is summarized in Table 2. Gravity environments are simulated as a downward acceleration in the z direction ($A_{z}$), so the expected $A_{z}$ is approximately −3.73 m/s^2^, −1.62 m/s^2^, and −0.000001 m/s^2^ for Martian, lunar, and microgravity, respectively. The $A_{z}$ values reported in Table 2 show the mean and standard deviation of the stable region of the parabola, during which the simulated gravity environment is most consistent even if it is not the region of the parabola that achieved the most accurate simulation of the target environment [20]. The standard deviation of this mean provides an insight into how well the environment is maintained. An accelerometer error caused only 15 parabolas to be recorded and is a limitation of this experiment. However, the Martian parabola that was recorded has the largest standard deviation in the acceleration experienced during the stable portion of the parabola, exceeding the standard deviation calculated for other parabolas by more than 0.1 m/s^2^. This supports the conclusion that the Martian gravity experiments had an additional variation which could influence the probe measurements.

**Figure 7:**
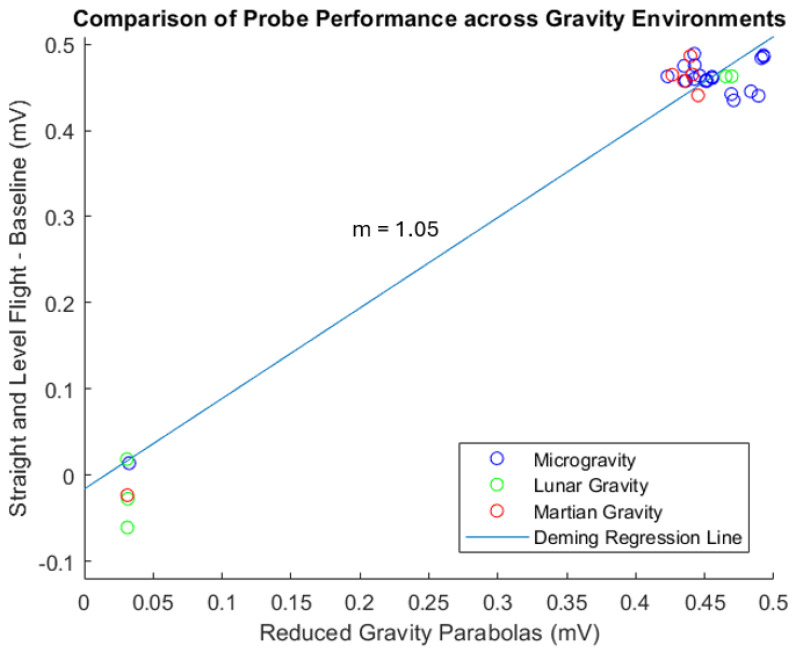
Probe performance is consistent across gravity environments. The two clusters represent an applied voltage of 0 V (lower group) and 5 V (upper group) to the phantom.

Deming regression is a statistical technique used to estimate bias when both the dependent and independent variables are measured with error [21]. This technique was applied to the data collected in-flight to evaluate the similarity between the probe measurements in 1 g and the reduced-gravity environments. K-means clustering was used to calculate the average distance from the centroid to quantify the variability in measurement within a cluster [22]. This was found to be 0.0224 and 0.0172 for the 0 V and 5 V clusters, respectively. This slight difference in variance can be attributed to the smaller sample size for the 0 V data points. The acceptance criteria for the use of Deming regression in method comparison studies require a slope close to 1; as shown in Figure 7, the slope across all reduced gravity parabolas was 1.05, indicating no presence of systemic bias in the probe’s measurement when operating in reduced gravity.

The PD probes record significantly different values when contact with the nasal cavity is not maintained. Benchtop testing of the probe and phantom system demonstrated a difference in measured PD values of approximately two orders of magnitude, and an order-of-magnitude reduction in signal noise when the probe was properly placed. This enables the easy identification of probe contact in the experiment. For example, Probe 1 became dislodged during the flight, during which the probe lost contact with the phantom wall; this was easily identified in the PD measurements. This conversely allows for confirmation that Probe 2 maintained contact throughout the experiment and ensures that all variations in measurement are a result of the experiment rather than contact fluctuations.

The results show the suitability of perfusion-based PD probes for operation in microgravity, lunar gravity, and hypergravity environments; the PD probe reached a technology readiness level (TRL) of 6 on NASA’s TRL scale in these environments [23].

### 5.2. Future Probe Design with Capillarity-Driven Perfusion

The perfusion pump included in the test setup (Figure 1) ensures consistent perfusion rates regardless of the gravity environment. However, Equation (3) shows that the perfusion tube operates under surface tension-dominated fluid behavior in Lunar, Martian, and microgravity. This indicates the possibility of designing a spaceflight-specific PD probe in which fluid perfusion is driven by capillary action in place of a perfusion pump, simplifying the PD system infrastructure and reducing both mass and volume requirements for launch.

In microgravity, the flow rate *Q* of fluid through a capillary can be calculated using the Washburn equation for fluid velocity [24] such that

(5) $$Q=\frac{\left(P_{A}+\frac{2\gamma }{r}\cos\phi \right)\pi r^{4}}{8\eta l}$$

where $P_{A}$ is the atmospheric pressure, γ and η are the surface tension and viscosity of the fluid, respectively, ϕ is the contact angle between the fluid and the tube, *l* is the length of the tube, and *r* is the tube radius. Assuming a tube length of 2 m, we can match the flow rate of 2.5 mL/h used in this experiment with r=0.08 mm. Commercial ultra-small tubes are available for r≥0.0635 mm [25], indicating the feasibility of the initial design. The Reynold’s number at the specified tube radius and fluid properties was calculated to be 4.68, which is well within the laminar flow regime.

Further investigation into the impacts of higher flow rates could enable the use of a larger *r*, and different capillary design techniques could also be applied to control flow rate [26]. Additional trade studies are needed to optimize tube radius, length, and material to achieve the desired flow rate and probe design for PD measurement, while mitigating the risk of tube bending or kink formation, which would prevent capillarity-driven flow. Other features, like adding an in-line bubble trap to the perfusion line, could address the propensity for bubble formation, which can disrupt probe contact with the measurement surface or introduce pressure differentials that reduce capillarity.

## 6. Conclusions

Perfusion-based PD probes were successfully demonstrated for use in spaceflight operation, reaching TRL 6 in microgravity, lunar gravity, and hypergravity environments. A novel nasal phantom was developed and used to mimic critical aspects of nasal physiology for rapid testing without human or animal subjects. This establishes the foundation for using probes that have a perfusion system in use in space, providing a step towards in-space biological testing. Future iterations of this work could include the development of a phantom that mimics the dynamic epithelial transport of live tissue, rather than the fixed ion concentration of the agar-based phantom used here. The results advance sensor capabilities in space to improve astronaut health.

## Figures and Tables

**Table 1:** *p*-value and effect size for Probe 2 data. *p*-value reports Wilcoxon rank sum test results, with significance criterion of *p* < 0.05. Results meeting the significance criterion are highlighted, indicating a statistically significant difference in the recorded measurement.

Test Pairing	5 V		0 V	
	***p*** **-Value**	**Effect Size**	***p*** **-Value**	**Effect Size**
Hypergravity vs. Baseline	0.5334	0.0057	0.2818	0.0247
Microgravity vs. Baseline	0.5636	0.0049	0.9048	0.0038
Lunar Gravity vs. Baseline	0.3810	0.0046	0.1000	0.0546
Martian Gravity vs. Baseline	0.0317	0.0254	0.5000	0.0546
Hypergravity vs. Microgravity	0.8380	0.0008	0.2238	0.0285
Hypergravity vs. Lunar Gravity	0.4644	0.0103	0.6000	0.0299
Hypergravity vs. Martian Gravity	0.0333	0.0197	1	0.0299
Microgravity vs. Lunar Gravity	0.4579	0.0010	0.1667	0.0584
Microgravity vs. Martian Gravity	0.0324	0.0205	0.5714	0.0584
Lunar Gravity vs. Martian Gravity	0.0952	0.0300	1	0.0002

**Table 2:** Achieved flight simulation of gravity environments.

Parabola Type and Index	Mean $A_{z}$ (m/s^2^)	Standard Deviation
Martian 1	−3.1780	0.3181
Lunar 1	−1.2722	0.1658
Lunar 2	−1.0320	0.0897
Lunar 3	−1.0990	0.0956
Microgravity 1	0.5166	0.1341
Microgravity 2	0.4632	0.1694
Microgravity 3	0.4627	0.1873
Microgravity 4	0.5309	0.0642
Microgravity 5	0.5154	0.0569
Microgravity 6	0.4872	0.0967
Microgravity 7	0.5540	0.0777
Microgravity 8	0.5542	0.1108
Microgravity 9	0.5981	0.1052
Microgravity 10	0.5051	0.0893
Microgravity 11	0.4800	0.1328

## Data Availability

The raw data supporting the conclusions of this article will be made available by the authors on request.

## Supplementary Materials

The following supporting information can be downloaded at: https://www.mdpi.com/article/10.3390/bios15080478/s1, Figure S1: Recorded PD variations in altered gravity environments in Probe 1; Table S1: Wilcoxon rank sum test results for Probe 1, with a significance criterion of *p* < 0.05.
