# Supplementary material for: Evaluating the Suitability of Perfusion-Based PD Probes for Use in Altered Gravity Environments
Source: Biosensors (Basel). 2025 Jul 24;15(8):478. doi: 10.3390/bios15080478 (PMC12384861; doi:10.3390/bios15080478)
Supplement: Supplementary file 1 [file biosensors-15-00478-s001.zip › biosensors-3672095-supplementary.pdf]

biosensors

MDPI

Article

Evaluating the Suitability of Perfusion-Based PD Probes for Use in Altered Gravity Environments

Madelyn MacRobbie [1,2,3,*], Vanessa Z. Chen [4], Cody Paige [5], David Otuya [3,6], Aleksandra Stankovic [1,6,7] and Guillermo Tearney [1,3,6,8]

1 Harvard-MIT Division of Health Sciences and Technology (HST), Boston, MA 02139, USA; stankov@mit.edu (A.S.); gtearney@mgb.org (G.T.)
2 Department of Aeronautics and Astronautics, Masschusetts Institute of Technology, Cambridge, MA 02139, USA
3 Wellman Center for Photomedicine, Massachusetts General Hospital, Boston, MA 02114, USA; dotuya@mgh.harvard.edu
4 Biomedical Engineering, University of Waterloo, Waterloo, ON N2L 3G1, Canada; vzchen@uwaterloo.ca
5 Space Exploration Initiative, MIT Media Lab, Cambridge, MA 02139, USA; cpaige@mit.edu
6 Harvard Medical School, Boston, MA 02115, USA
7 Center for Space Medicine Research, Massachusetts General Hospital, Boston, MA 02114, USA
8 Department of Pathology, Massachusetts General Hospital, Boston MA 02114, USA
* Correspondence: hoyingm@mit.edu

check for updates

Received: 14 May 2025
Revised: 3 July 2025
Accepted: 14 July 2025
Published:

Citation: MacRobbie, M.; Chen, V.Z.; Paige, C.; Otuya, D.; Stankovic, A.; Tearney, G. Evaluating the Suitability of Perfusion-Based PD Probes for Use in Altered Gravity Environments. *Biosensors* **2025**, *1*, 0. https://doi.org/

Copyright: © 2025 by the authors. Licensee MDPI, Basel, Switzerland. This article is an open access article distributed under the terms and conditions of the Creative Commons Attribution (CC BY) license (https://creativecommons.org/licenses/by/4.0/).

Test Pairing	Probe 1, 5V	Probe 1, 0V
Hypergravity vs Baseline	0.6429	0.5714
Microgravity vs Baseline	0.1905	0.8571
Hypergravity vs Microgravity	0.0823	0.8413

Table S1. Wilcoxon rank sum test results for Probe 1, with significance criterion of $p < 0.05$.

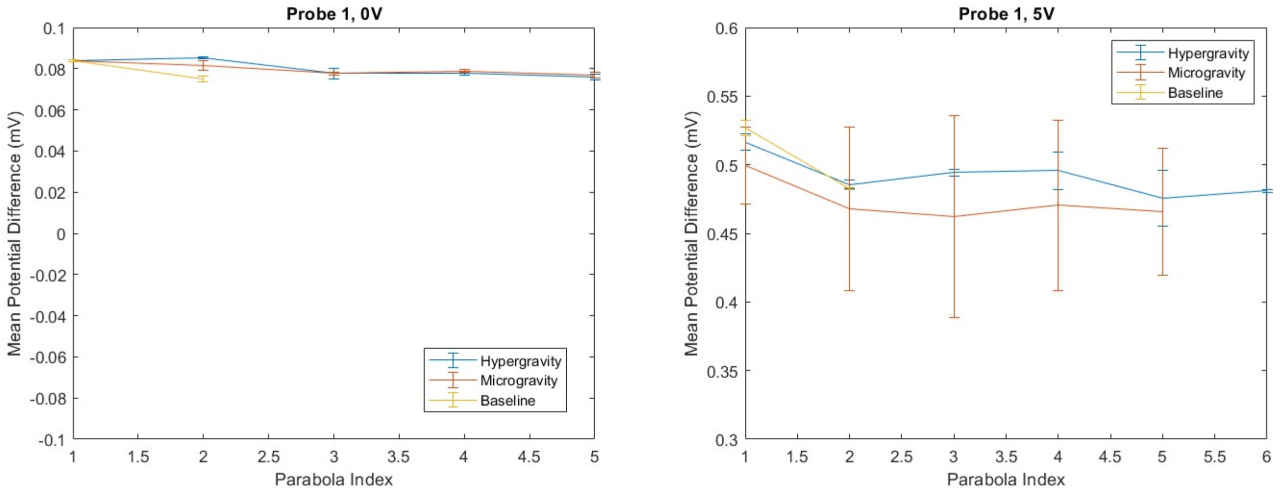

(**a**) (**b**)

Figure S1. Recorded PD variations in altered gravity environments in Probe 1.
